# Pathway-Based Genome-Wide Association Analysis Identified the Importance of Regulation-of-Autophagy Pathway for Ultradistal Radius BMD

**DOI:** 10.1002/jbmr.36

**Published:** 2010-01-29

**Authors:** Lishu Zhang, Yan-Fang Guo, Yao-Zhong Liu, Yong-Jun Liu, Dong-Hai Xiong, Xiao-Gang Liu, Liang Wang, Tie-Lin Yang, Shu-Feng Lei, Yan Guo, Han Yan, Yu-Fang Pei, Feng Zhang, Christopher J Papasian, Robert R Recker, Hong-Wen Deng

**Affiliations:** 1 Institute of Bioscience and Biotechnology, School of Science, Beijing Jiaotong UniversityBeijing, People's Republic of China; 2 Departments of Orthopedic Surgery and Basic Medical Sciences, University of Missouri–Kansas CityKansas City, MO, USA; 3 The Key Laboratory of Biomedical Information Engineering, Ministry of Education and Institute of Molecular Genetics, School of Life Science and Technology, Xi'an Jiaotong UniversityXi'an, People's Republic of China; 4 Osteoporosis Research Center, Creighton UniversityOmaha, NE, USA

**Keywords:** osteoporosis, bone mineral density, genome-wide association, regulation of autophagy, whites

## Abstract

Wrist fracture is not only one of the most common osteoporotic fractures but also a predictor of future fractures at other sites. Wrist bone mineral density (BMD) is an important determinant of wrist fracture risk, with high heritability. Specific genes underlying wrist BMD variation are largely unknown. Most published genome-wide association studies (GWASs) have focused only on a few top-ranking single-nucleotide polymorphisms (SNPs)/genes and considered each of the identified SNPs/genes independently. To identify biologic pathways important to wrist BMD variation, we used a novel pathway-based analysis approach in our GWAS of wrist ultradistal radius (UD) BMD, examining approximately 500,000 SNPs genome-wide from 984 unrelated whites. A total of 963 biologic pathways/gene sets were analyzed. We identified the regulation-of-autophagy (ROA) pathway that achieved the most significant result (*p* = .005, *q*_*fdr*_ = 0.043, *p*_*fwer*_ = 0.016) for association with UD BMD. The ROA pathway also showed significant association with arm BMD in the Framingham Heart Study sample containing 2187 subjects, which further confirmed our findings in the discovery cohort. Earlier studies indicated that during endochondral ossification, autophagy occurs prior to apoptosis of hypertrophic chondrocytes, and it also has been shown that some genes in the ROA pathway (e.g., *INFG*) may play important roles in osteoblastogenesis or osteoclastogenesis. Our study supports the potential role of the ROA pathway in human wrist BMD variation and osteoporosis. Further functional evaluation of this pathway to determine the mechanism by which it regulates wrist BMD should be pursued to provide new insights into the pathogenesis of wrist osteoporosis. © 2010 American Society for Bone and Mineral Research.

## Introduction

Osteoporosis (MIM 166710) is a systemic skeletal disease characterized by low bone mass and microarchitectural deterioration of bone tissue with a consequent increase of bone fragility and susceptibility to fractures.(1) Osteoporosis has become one of the most serious public health problems around the world, causing millions of fractures annually.(2) Fractures at the distal radius of the wrist, also known as *Colles' fractures*, are the most common fractures in women younger than 75 years of age.(3) Most Colles' fractures are the combined result of a low-energy fall and loss of wrist bone mass owing to osteoporosis. Bone mineral density (BMD) at the distal forearm is the main determinant of susceptibility to Colles' fracture.(4)

BMD, the proxy measure for osteoporosis, has a strong genetic determination. The results of twin and family studies indicate that genetics account for 60% to 90% of the variance in BMD at multiple skeletal sites, including the wrist.(5) BMD variation at different skeletal sites has been shown to share some genetic factors.(6) Since wrist fractures were found to be predictive for fractures at other skeletal sites,(7–9) the wrist, especially the distal radius, may be a useful site for early assessment of the overall osteoporotic fracture risk.

With the emergence of genome-wide linkage disequilibrium (LD)–based marker panels and the improvements in high-throughput genotyping technology, genome-wide association studies (GWASs) have become feasible. GWASs can systematically survey the whole genome for causal genetic variants for complex traits/diseases and are powerful tools for dissecting the genetic basis for osteoporosis. Several promising candidate genes for osteoporosis or related traits have been identified by GWASs.(10–13)

Most published GWASs consider the effects of genetic markers or haplotypes independently and focus only on genes or markers of top-ranking statistical significance. Genes and/or their products often work together, however, interacting in functional groups or pathways to contribute to phenotypic variation or susceptibility to a disease. Therefore, the approach just outlined, which focuses on individual genes, may not be optimally effective for identifying pathophysiologically significant genetic pathways underlying complex traits or diseases such as osteoporosis. Recently, motivated by the availability of gene set enrichment analysis (GSEA) for gene expression data, Wang and colleagues(14) developed a pathway-based genome-wide association (GWA) analysis method that uses up-to-date knowledge of functional interactions among genes in biologic pathways. With this method, multiple genes in a related pathway can be considered jointly, and association with a group of genes as a whole (pathway) can be detected efficiently, even though some individual genes may have only moderate association evidence. The advantage of this approach was illustrated in its application to two Parkinson disease (PD) GWA data sets and one amyotrophic lateral sclerosis (AMD) data set.(14)

To identify novel biologic pathways contributing to wrist osteoporosis and Colles' fractures, we performed a pathway-based GWA analysis on BMD variation at the wrist ultradistal radius (UD) using the method proposed by Wang and colleagues.(14) We identified a significant association, at the pathway-wise level, between UD BMD and the regulation-of-autophagy (ROA) pathway. Our findings support the potential role of the ROA pathway in the pathogenesis of wrist osteoporosis.

## Materials and Methods

### Subjects

*Discovery GWAS sample*: This study was approved by the necessary Institutional Review Boards of all the involved institutions. A total of 984 unrelated US whites of European origin were chosen from our established genetic repertoire currently containing more than 10,000 subjects. Signed informed-consent documents were obtained from all study participants before they entered the study.

People with chronic diseases and conditions that potentially might affect bone mass, structure, or metabolism were excluded from the study. These diseases/conditions included chronic disorders involving vital organs (eg, heart, lung, liver, kidney, and brain), serious metabolic diseases (eg, diabetes, hypo- and hyperparathyroidism, hyperthyroidism, etc.), other skeletal diseases (eg, Paget disease, osteogenesis imperfecta, rheumatoid arthritis, etc.), chronic use of drugs affecting bone metabolism (eg, hormone-replacement therapy, corticosteroid therapy, and anticonvulsant drugs), and malnutrition conditions (eg, chronic diarrhea, chronic ulcerative colitis, etc.). In addition, subjects taking anti-bone-resorptive or bone anabolic agents/drugs such as bisphosphonates were excluded from the study.

For all subjects, measurement of anthropometric variables was performed, and a structured questionnaire addressing lifestyles and medical history was administered. Areal BMD (g/cm^2^) at the UD region of the wrist was measured by dual-energy X-ray absorptiometry (DXA) with Hologic QDR 4500W densitometers (Hologic, Inc., Bedford, MA, USA). The scanners were calibrated daily, and long-term precision was monitored with external phantoms. The coefficient of variation (*CV*%) of wrist UD BMD measurements obtained on the Hologic scanner was approximately 2.3%.

*FHS replication sample*: For replication of our GWAS findings, we used a sample from the Framingham Heart Study (FHS). The FHS is a community-based, multigenerational, longitudinal study of cardiovascular disease and its risk factors, with one substudy focusing on osteoporosis. The phenotype and genotype information of the cohort was downloaded from the Framingham SNP Health Association Resource (SHARe), accessed through the database of Genotypes and Phenotypes (dbGaP) developed by the National Center for Biotechnology Information (NCBI) (http://view.ncbi.nlm.nih.gov/dbgap). Appropriate procedures have been taken for the use of the data, which include approval from the University of Missouri–Kansas City Institutional Review Board and signatures on the Data Distribution Agreement by all the UMKC investigators who have access to the data.

The FHS comprises the original cohort, offspring, and generation 3 studies. Subjects used as the replication sample in this analysis are those who participated in exam 23 of the first generation and exams 6 and 7 of the second generation with arm BMD as well as other covariates, including age, weight, height, and sex, available. The arm BMD was scanned by DXA (Lunar DPX-L, Lunar Co., Madison, WI, USA). The detailed protocol is available through the NCBI dbGaP. In total, the sample contains 2187 subjects (817 males and 1370 females) from 477 families.

### Genotyping

*Discovery GWAS sample*: Genomic DNA was extracted from whole human blood using a commercial isolation kit (Gentra Systems, Minneapolis, MN, USA) following the protocols detailed in the kit. DNA concentration was assessed by a DU530 UV/VIS spectrophotometer (Beckman Coulter, Inc., Fullerton, CA, USA). Genotyping with the Affymetrix Mapping 250K Nsp and Affymetrix Mapping 250K Sty arrays was performed at the Vanderbilt Microarray Shared Resource at Vanderbilt University Medical Center (Nashville, TN, USA) using the standard protocol recommended by the manufacturer (Affymetrix, Inc., Santa Clara, CA, USA). Briefly, for each array, 250 ng of genomic DNA was digested with either Nsp1 or Sty1 and ligated to adapters that recognize the cohesive 4-base-pair (bp) overhangs. A generic primer that recognizes the ligated adapter sequence was used to amplify the ligation products in a polymerase chain reaction (PCR). The amplified DNA was assayed by agarose gel electrophoresis to verify an average size distribution of 250 to 1500 bp. The amplified DNA was purified per the manufacturer's protocol and quantified by absorbance at 260 and 280 nm. Then 90 µg of purified DNA was fragmented with DNase I and visualized on a 4% agarose gel. Samples with fragment distributions of less than 180 bp were hybridized to the appropriate array (Nsp or Sty). Arrays were stained, washed, and scanned per manufacturer's protocol using immunopure strepavidin (Pierce, Milwaukee, WI, USA), biotinylated antistreptavidin antibody (Vector Labs, Burlingame, CA, USA), and *R*-phycoerythrin strepavidin (Invitrogen, Carlsbad, CA, USA). Fluorescence intensities were quantitated using an Affymetrix Array Scanner 30007G. Data management and analyses were performed using the Affymetrix GeneChip Operating System. Genotyping calls were determined from the fluorescent intensities using the Dynamic Model-based (DM) algorithm with a 0.33 confidence score setting,(15) as well as the Robust Linear Model with Mahalanobis Distance Classifier with a Bayesian step (B-RLMM) algorithm.(16) According to Affymetrix's guidelines, DM calls were used for quality control. Specifically, subjects with DM call rates of less than 93% were subject to regenotyping. Finally, 99% of all the subjects passed this quality-control criterion, and the average call rate for all the subjects reached greater than 95%. The B-RLMM algorithm was used for the association analyses owing to its improved performance.(16) B-RLMM clustering was performed with 94 samples per cluster.

The final average B-RLMM call rate across the entire sample in this study reached the high level of 98.57%. However, of the initial full set of 500,568 SNPs, we discarded 32,961 SNPs with per-SNP call rates (ie, call rate for each SNP across all samples) of less than 95% and another 36,965 SNPs with allele frequencies deviating from Hardy-Weinberg equilibrium (HWE; *p* < .001). We further discarded SNPs with a minor allele frequency (MAF) of less than 5% and SNPs that are more than 500 kilobases (kb) away from any gene. The physical distance of 500 kb was set because most enhancers and repressors are less than 500 kb away from genes, and most LD blocks are less than 500 kb. In total, 312,172 SNPs, which covered 14,585 genes, were assayed in the following pathway-based GWA analyses.

*FHS replication sample*: Genotyping in the FHS sample was carried out with the Affymetrix 500K mapping array plus the Affymetrix 50K supplementary array. For details of the genotyping method, please refer to the Framingham SHARe at the NCBI dbGaP Web site (http://www.ncbi.nlm.nih.gov/projects/gap/cgi-bin/study.cgi?study_id=phs000007.v3.p2).

### Generation of pathway/gene sets collection

We used four different resources to generate a collection of annotated gene sets and pathways to be tested in this study, which are the BioCarta pathway database (http://www.biocarta.com/genes), the KEGG pathway database (http://www.genome.ad.jp/kegg/pathway.html), the Ambion GeneAssist Pathway Atlas (http://www.ambion.com/tools/pathway), and the Gene Ontology (GO) database (http://www.geneontology.org). A total of 260, 190, and 380 annotated pathways were retrieved from the BioCarta pathway database, KEGG pathway database, and Ambion GeneAssist Pathway Atlas, respectively. To integrate the GO information into our study, GO annotation files for human genes also were downloaded. We processed the GO annotation files and generated gene sets on the basis of GO level 4 annotations in biologic process and molecular function. Genes whose GO annotations are in level 5 or lower in the GO hierarchy are assigned to their ancestral GO annotations in level 4. In our analysis, we tested only pathways/gene sets in which at least 85% (and in number 10 to 200) of the genes were included in our GWA data set so as to alleviate the multiple-testing problem by avoiding testing too narrowly or too broadly defined functional categories. Overall, 963 pathways/gene sets were analyzed.

### Statistical analysis

*Analyses in the discovery sample*: To detect population stratification that may lead to spurious association results, we used Structure 2.2 software (http://pritch.bsd.uchicago.edu/software.html) and EIGENSOFT 2.0 software (http://genepath.med.harvard.edu/~reich/EIGENSTRAT.htm) to investigate the potential substructure/stratification of our sample. The Structure 2.2 program uses a Markov chain Monte Carlo (MCMC) algorithm to cluster individuals into different cryptic subpopulations on the basis of multilocus genotype data.(17) Using the software, we performed independent analyses under three assumed numbers of population strata (*k* = 2, 3, and 4) using 200 unlinked, randomly selected markers. To confirm the results achieved through Structure 2.2, we further tested population stratification in our sample using EIGENSOFT 2.0 software, which uses both principal-component analysis and a genomic control approach to correct possible statistical bias caused by ancestral differences in whole-genome association studies.(18,19)

Routine GWA statistical analyses on individual SNPs were first conducted by the Wald test implemented in Plink (Version 1.03, http://pngu.mgh.harvard.edu/~purcell/plink) to obtain a *t* statistic for each tested SNP.(20) The parameters, including age, age^2^, sex, age/age^2^-by-sex interaction, height, and weight, were tested for their associations with UD BMD. The significant (*p* ≤ .05) terms (age, age^2^, sex, and weight) then were included as covariates to adjust the raw UD BMD values for subsequent analyses. The adjusted UD BMD data were normally distributed (*p* > .1).

Subsequently, pathway-based GWA analysis was performed following the approach developed by Wang and colleagues.(14) Briefly, the main procedures are as follows:

1. *Generation of statistic value of gene-phenotype association*: Suppose *N* genes in the whole genome and each gene with *x*_*i*_ (*i* ≤ *N*) SNPs mapped to it. For a specific gene *G*_*i*_, the highest SNP-phenotype association statistic value among all *x*_*i*_ SNPs was selected to represent the statistic value of the gene, denoted as *r*_*i*_. Mostly, SNPs were mapped to their closest gene. And in rare cases, if an SNP was located within shared regions of two overlapping genes, the SNP was mapped to both genes.
2. *Ranking statistic values of gene-phenotype association*: We ranked all genes by sorting their statistic values from the largest to smallest, denoted as gene list *L* (*r*_1_, *r*_2_, …, *r*_*N*_).
3. *Enrichment score calculation*: For each given pathway/gene set *S*, composed of *N*_*S*_ genes, a statistic enrichment score (*ES*) was calculated. *ES* is a weighted Kolmogorov-Smirnov–like running-sum statistic that reflects the overrepresentation of genes within *S* at the top of the entire ranking list of genes in the genome. The score is calculated by walking down the gene list *L*, increasing a running-sum statistic when we encounter a gene in *S* and decreasing it when we encounter genes not in *S*. *ES* is the maximum deviation from zero encountered in the random walk.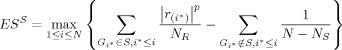  where 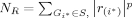, and *p*(designated as 1 here) is a parameter that gives higher weight to genes with extreme statistic values. Thus, both the rank of each gene in the entire ranked gene list L (*N*_*S*_) and the association statistic value of it (*r*_*i*_) will contribute to the *ES* value of a pathway.
4. *Permutation and nominal significance assessment*: To estimate the significance level of *ES*, permutation procedures were used to create the null distribution of *ES* for each pathway/gene set. The phenotype data of sampled subjects were first shuffled. Then the previous three steps were repeated to calculate *ES* for the pathway/gene set during each permutation. In total, 1000 permutations were done. Finally, a distribution for *ES* 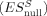 was generated for each pathway/gene set *S*. The significance of an observed *ES*^*S*^ for a pathway/gene set (nominal *p* value) was estimated as the percentage of permutations whose 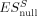 values were greater than the observed *ES*^*S*^.
5. *Multiple-testing adjustments*: To compare the significance among pathways with different numbers of genes, first, a normalized*ES*, (*NES*) was constructed based on the observed *ES*, mean and standard deviation(SD) of 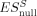.

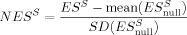


Then two measures were used to adjust for multiple-hypothesis testing for more reliable results. False-discovery rate (FDR) is a procedure usually used to control the fraction of expected false-positive findings to stay below a certain threshold. Family-wise error rate (FWER) is another approach to correct for multiple-hypotheses testing that is considered to be a highly conservative procedure seeking to ensure that the list of reported results does not include even a single false-positive gene set.(14,21) For a pathway/gene set, let 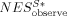 denote the normalized enrichment score in the observed data. The FRD *q* value (denoted as 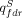) was calculated as the ratio of the fraction of all permutations with *NES* ≥ 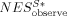 to the fraction of observed pathways/gene sets with *NES* ≥ 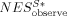. FWER *p* value (denoted as 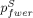) was calculated as the fraction of pathways/gene sets whose greatest *NES* among all permutations is greater than 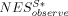. In this study, the significance criteria after multiple-testing correction for a pathway is that both FDR *q* value and FWER *p* value are .05 or less.


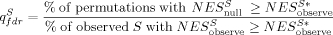



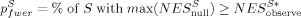


*Replication analyses with FHS sample*: The statistical analyses for the FHS sample were almost the same as those in the discovery GWAS cohort unless some little modifications regarding that FHS is a family-based study. In brief, first, the raw arm BMD data were adjusted. The parameters, including age, age^2^, sex, age/age^2^-by-sex interaction, height, and weight, were tested for their associations with arm BMD. The significant (*p* ≤ .05) terms (age-by-sex, height, and weight) then were included as covariates to adjust the raw arm BMD values for subsequent analyses. The adjusted UD BMD data were normally distributed (*p* > .1). Second, the routine GWA statistical analyses on individual SNPs were conducted using FBAT software (http://biosun1.harvard.edu/~fbat/fbat.htm)(22) to obtain a *Z* statistic for each tested SNP. Third, a pathway-based GWA analysis was performed with almost the same method as that in the discovery cohort unless that the *Z* statistical values for all genes other than the phenotype data were shuffled when doing the permutation and nominal significance assessment so that the family structure of the population could be maintained.(14) A total of 10,000 permutations were done.

## Results

### Characteristics of study subjects

In the discovery GWAS cohort, a total of 984 individuals were included in the analyses. The basic characteristics of all subjects in this cohort, stratified by sex, are shown in Table 1. The plot of the UD BMD *T*-scores (mean = −0.53, SD = 2.01) in this population are given in Supplemental Fig. S1. There are 2187 subjects (817 males and 1370 females) from 477 families in the FHS who were studied to replicate the findings from the discovery cohort. The basic characteristics of all subjects in the FHS sample, stratified by sex, are shown in Table 2.

**Table 1:** Basic Characteristics of the Discovery Cohort^a^

Trait	Male (*n* = 489)	Female (*n* = 495)
Age (years)	50.60 (18.87)	50.39 (17.65)
Height (cm)	177.89 (6.96)	163.83 (6.52)
Weight (kg)	89.00 (14.96)	71.40 (15.97)
UD BMD (g/cm^2^)	0.507 (0.073)	0.418 (0.065)

a The data are mean (SD) of raw values.

**Table 2:** Basic Characteristics of FHS Subjects^a^

Trait	Male (*n* = 817)	Female (*n* = 1370)
Age (years)	63.84 (11.49)	63.95 (11.74)
Height (inch)	68.31 (2.80)	62.94 (2.71)
Weight (pounds)	176.85 (22.44)	146.93 (25.78)
Arm BMD (g/cm^2^)	0.983 (0.097)	0.784 (0.098)

a The data are mean (SD) of raw values.

### Pathway-based association analyses in the discovery cohort

No significant population stratification was detected in the discovery cohort (984 subjects) by either Structure 2.2(17) or EIGENSOFT 2.0 software.(18) When using Structure 2.2 software, all subjects were tightly clustered together, suggesting no population stratification (data not shown). The results from EIGENSOFT 2.0 software(18) provided further support for the results from Structure 2.2. Only one principal component was significant in the principal-component analysis (*p* < .001), suggesting that only one population ancestry existed for our sample. Based on genome-wide SNP information, inflation factor λ, a measure of population stratification, also was estimated by the genomic control(19) approach implemented in EIGENSOFT 2.0 software. Ideally, for a homogeneous population with no stratification, the value of the inflation factor parameter λ should be equal to or near 1. In our study, λ = 1.01, indicating that the sample population is homogeneous.

**Fig. 1:**
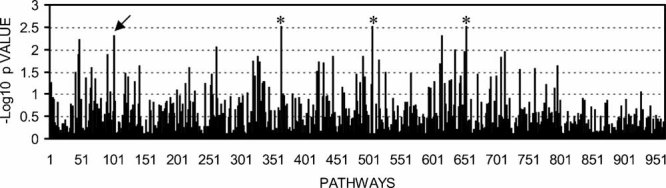
–Log10 *p* values of all the 963 pathways with UD BMD. The arrow points to the ROA pathway. It should to be noted that there are three pathways (marked with *) whose nominal *p* values were less than that of the ROA pathway. However, after multiple-testing correction, these three pathways do not meet the significance criteria, that is, both *FDR* and *FWER* ≤ 0.05.

**Fig. 2:**
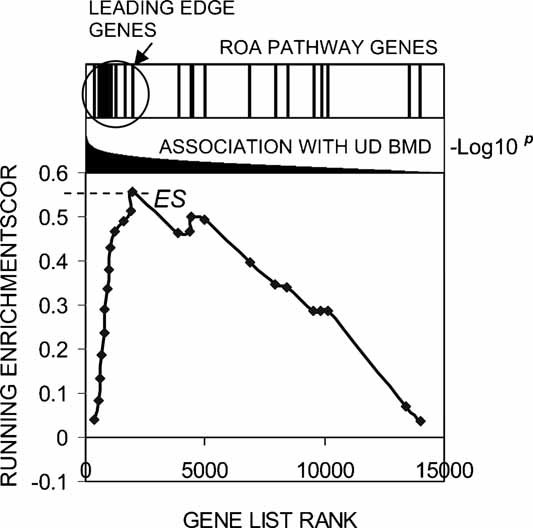
Running-sum plot for the ROA pathway, including the location of the maximum enrichment score (*ES*) and the leading-edge subset. The *x* axis is the rank of the 25 genes in the ROA pathway in the whole gene list generated by ranking all the genes by their association significance with wrist BMD from the largest to smallest. These 25 genes are, from left to right, *IFNA14*, *ATG12*, *IFNA21*, *IFNA5*, *IFNA17*, *IFNA8*, *IFNA16*, *IFNA4*, *IFNA7*, *ATG7*, *IFNA13*, *PIK3C3*, *ATG5*, *IFNG*, *IFNA1*, *GABARAPL1*, *IFNA2*, *ULK2*, *ATG3*, *IFNA10*, *BECN1*, *PRKAA2*, *PIK3R4*, *IFNA6*, and *PRKAA1*. The *y* axis represents the running enrichment score. *ES* is the maximum deviation from zero achieved in the running-sum walk. Owing to a large number of genes ranked at the top of the whole gene list genome-wide, this pathway achieved a high *ES* (*ES* = 0.557). The bar plot at the top of the figure also shows the distribution of the 25 genes from the ROA pathway in the whole gene list, with the leading-edge genes indicated.

Among all 963 pathways tested in our analysis, the ROA pathway, which contains 25 genes, showed the strongest significant association with wrist UD BMD. The ROA pathway achieved a high *ES* of 0.557 with a *p* value of .005 (Figs. 1 and 2). Most of the genes in the ROA pathway ranked at the top in significance in the gene list containing 14,585 genes genome-wide, with 13 genes ranked among the top 2000 genes (Fig. 2). More important, it is the only pathway, among all 963 pathways tested, that achieved statistically significant *FDR* and *FWER* values (*NES* = 2.50, *q*_*fdr*_ = 0.043, *p*_*fwer*_ = 0.016).

The ROA pathway is annotated by the KEGG database. It is the only pathway related to autophagy among all the pathways tested. As shown in Table 1, the ROA pathway contains 6 genes (*ATG12*, *ATG3*, *ATG5*, *ATG7*, *BECN1*, and *GABAPAPL1*) encoding essential proteins involved directly in different stages of autophagy, for example, formation and elongation of phagophores and autophagosomes.(23,24) In addition, it contains 14 genes encoding type I and type II interferons (IFNs), which are pleiotropic cytokines that contribute to regulation of autophagy.(25,26) The pathway also contains five genes encoding subunits of kinases, which are autophgy modulators. These kinases include the catalytic subunit and regulatory subunit of lipid kinase class III phosphoinositide 3-kinase (PIK3C3), two catalytic subunits of AMP-activated protein kinase (AMPK), and unc-51-like kinase (ULK2). The individual gene with the most significant *p* value among all the genes in this pathway is *IFNA14* (*p* = 1.10 × 10^−3^). Other genes in the ROA pathway that also contribute positively to the *ES* (ie, the genes that ranked before or at the point of *ES*, also denoted as *leading-edge genes*) are *ATG12* (*p* = 2.11 × 10^−3^), *IFNA21* (*p* = 2.90 × 10^−3^), *IFNA5* (*p* = 3.16 × 10^−3^), *IFNA17* (*p* = 3.49 × 10^−3^), *IFNA8* (*p* = 4.92 × 10^−3^), *IFNA16* (*p* = 5.46 × 10^−3^), *IFNA4* (*p* = 6.02 × 10^−3^), *IFNA7* (*p* = 6.55 × 10^−3^), *ATG7* (*p* = 8.32 × 10^−3^), *IFNA13* (*p* = 1.21 × 10^−2^), *PIK3C3* (*p* = 1.57 × 10^−2^), and *ATG5* (*p* = 1.62 × 10^−2^) (Table 3).

**Table 3:** Genes in Regulation-of-Autophagy (ROA) Pathway

Gene symbol	Gene ID	Genome location	Full name
*ATG12*	9140	5q21-q22	ATG12 autophagy-related 12 homologue (*S. cerevisiae*)
*ATG3*	64422	3q13.2	ATG3 autophagy-related 3 homologue (*S. cerevisiae*)
*ATG5*	9474	6q21	ATG5 autophagy-related 5 homologue (*S. cerevisiae*)
*ATG7*	10533	3p25.3-p25.2	ATG7 autophagy-related 7 homologue (*S. cerevisiae*)
*BECN1*	8678	17q21	Beclin 1, autophagy-related. (also known as *ATG6*)
*GABARAPL1*	23710	12p13.2	GABA(A) receptor–associated protein like 1 (*ATG8*)
*IFNA1*	3439	9p22	Interferon, α1
*IFNA10*	3446	9p22	Interferon, α10
*IFNA13*	3447	9p22	Interferon, α13
*IFNA14*	3448	9p22	Interferon, α14
*IFNA16*	3449	9p22	Interferon, α16
*IFNA17*	3451	9p22	Interferon, α17
*IFNA2*	3440	9p22	Interferon, α2
*IFNA21*	3452	9p22	Interferon, α21
*IFNA4*	3441	9P22	Interferon, α4
*IFNA5*	3442	9p22	Interferon, α5
*IFNA6*	3443	9p22	Interferon, α6
*IFNA7*	3444	9p22	Interferon, α7
*IFNA8*	3445	9p22	Interferon, α8
*IFNG*	3458	12q14	Interferon, γ
*PIK3C3*	5289	18q12.3	Phosphoinositide-3-kinase, class3 (*VPS34*)
*PIK3R4*	30849	3q22.1	Phosphoinositide-3-kinase, regulatory subunit 4 (*VPS15*)
*PRKAA1*	5562	5p12	AMP-activated protein kinase (AMPK) α1 catalytic subunit
*PRKAA2*	5563	1p31	AMP-activated protein kinase (AMPK) α2 catalytic subunit
*ULK2*	9706	17p11.2	Unc-51-like kinase.

Information for the SNPs whose *p* values were selected to represent the *p* values of the 13 leading-edge genes is given in Table 4. All the SNPs are common variants with minor allele frequencies (MAF) of 8% or greater in our sample. We used the FASTSNP program (http://fastsnp.ibms.sinica.edu.tw) to analyze potential functions of these SNPs. The SNP rs7037147 is located in the intron of *IFNA14* and is suggested as an intronic enhancer. An A-to-G change at this locus will delete a binding site of the transcription factor *SRY*. The SNP rs10757219 is located at a transcription factor binding site at the upstream regulatory region of *IFNA8*. An A-to-G change at this locus will delete a binding site of the transcription factor *c-Myc*.

**Table 4:** The Most Significant SNPs Mapped to the Leading-Edge Genes of the ROA Pathway

SNP	Gene	*p* Value	Role	Allele	MAF^a^
rs7037147	*IFNA14*	1.10 × 10^−3^	Intron	A/G	0.16
rs6883910	*ATG12*	2.11 × 10^−3^	Upstream	A/C	0.39
rs7852323	*IFNA21*	2.90 × 10^−3^	Upstream	A/G	0.17
rs1360286	*IFNA5*	3.16 × 10^−3^	Downstream	A/C	0.21
rs10964938	*IFNA17*	3.49 × 10^−3^	Upstream	A/C	0.18
rs10757219	*IFNA8*	4.92 × 10^−3^	Promoter/regulatory region	A/G	0.28
rs2015345	*IFNA16*	5.46 × 10^−3^	Downstream	A/C	0.17
rs1477479	*IFNA4*	6.02 × 10^−3^	Downstream	A/T	0.17
rs7856345	*IFNA7*	6.55 × 10^−3^	Downstream	A/T	0.18
rs17536147	*ATG7*	8.32 × 10^−3^	Intron	C/G	0.08
rs637949	*IFNA13*	1.21 × 10^−2^	Upstream	C/G	0.17
rs9955346	*PIK3C3*	1.57 × 10^−2^	Upstream	A/G	0.17
rs694433	*ATG5*	1.62 × 10^−2^	Upstream	A/G	0.21

a MAF (minor allele frequency) in our sample.

### Replication study in the FHS sample

To further confirm our GWAS findings in the discovery cohort, we carried out a replication analysis with a sample from the FHS population, which contains 2187 subjects. Same as that in the discovery cohort, 963 pathways were analyzed. In total, this analysis covered 15,598 genes genome-wide. All 25 genes of the ROA pathway, as shown in Table 3, were included for association testing. For the ROA pathway, the observed *ES* is 0.24, observed *NES* = 13.77; based on 10,000 permutation, nominal *p* value < 10^−4^, *q*_*fdr*_ = 0.001, and *p*_*fwer*_ = 0.007. That is, the ROA pathway showed significant association with arm BMD in this FHS sample even after multiple-testing adjustment. The leading-edge genes of the ROA pathway in the replication FHS cohort are *PIK3C3* (*p* = 1.97 × 10^−3^), *ATG12* (*p* = 8.16 × 10^−3^), *PRKAA2* (*p* = 8.28 × 10^−3^), *ATG5* (*p* = 1.45 × 10^−2^), *GABARAPL1* (*p* = 3.44 × 10^−2^), *BECN1* (*p* = 4.45 × 10^−2^), *IFNA13* (*p* = 4.72 × 10^−2^), and *ATG7* (*p* = 4.92 × 10^−2^), five of which (ie, *PIK3C3*, *ATG12*, *ATG5*, *IFNA13*, and *ATG7*) are also leading-edge genes in the discovery cohort. Therefore, there is significant overlapping in the genes involved between the discovery and replication cohorts that contribute to the association signals at the ROA pathway level. The information of the most significant SNPs mapped to these leading-edge genes in the replication FHS cohort are presented in Supplemental Table S1.

## Discussion

In this study, a genome-wide association analysis was conducted to identify the pathway(s) involved in BMD determination at a clinically important skeletal site—wrist UD. The ROA pathway was shown to be significantly associated with wrist UD BMD (*p* = .005) and was the only pathway, among a total of 963 pathways tested, that achieved statistically significant *FDR* and *FWER* values. To further confirm our findings in the discovery cohort, 2187 subjects from the FHS with arm BMD data available were studied as a replication study. The ROA pathway showed significant association with arm BMD in the FHS sample even after the multiple-testing adjustment, which further confirmed our findings in the discovery cohort. This is the first time that the autophagy-related biologic process has been implicated as an underlying factor for wrist BMD variation and hence a risk factor for wrist osteoporosis.

**Fig. 3:**
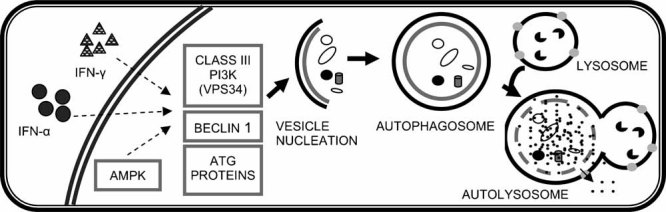
Autophagy and the functional interactions among the genes in the ROA pathway. Basically, the autophagic process can be divided into three stages: vesicle nucleation, vesicle elongation and completion, and vesicle breakdown and degradation. ATG1, ULK, ATG6 (Beclin1), PIK3R4 (VPS15), and lipid kinase PIK3C3 (VPS34) are key proteins that mediate vesicle nucleation. AMPK and IFNs are important upstream regulators of autophagy. Many other ATG proteins, such as ATG3, ATG5, ATG7, ATG8, and ATG12, play important roles in elongation of the phagophore and formation of the autophagosome.

Autophagy is a conserved housekeeping function of eukaryotic cells that permits sequestration of cytoplasmic components in membrane-bound vesicles and delivers them to lysosomes for degradation. Autophagy is subject to suppression or further induction in response to different stresses, starvation, specific hormonal regulation, and other stimuli.(24) The core process of autophagy and the functional interactions among the genes in the ROA pathway are depicted in Fig. 3. The autophagy pathway has numerous proposed physiologic functions. Growing evidence suggests that malfunctions of autophagy contribute to many human diseases, for example, myopathies, liver disease, neurodegeneration, heart disease, and cancer.(23,24)

Although this article is the first to support the ROA pathway's importance to osteoporosis, the pathway's relevance to bone metabolism has been suggested in previous studies. Autophagy has been recognized as an intermediate stage during the terminal differentiation of chondrocytes. Specifically, during endochondral ossification, induction of autophagy occurs prior to apoptotic hypertrophic maturation in terminally differentiated chondrocytes.(27,28) These chondrocytes morphologically exhibit autophagic characteristics and express autophagic proteins.(27,29) The induction of autophagy enhances the survival of chondrocytes in hypoxic microenvironments (eg, low protein, glucose, and O_2_ concentrations) and facilitates synthesis of the calcified extracellular matrix.(30–32)

Some genes in the ROA pathway also have been considered as important modulating factors for bone development or remodeling. For example, IFN-α can suppress the proliferation of osteoprogenitor cells(33,34) and modify the expression of a number of important cytokines that are regulators for human osteoprogenitor cell growth and differentiation.(35–37) IFN- α also can inhibit the differentiation of peripheral blood mononuclear cells (PBMCs) to osteoclasts.(38) Interferon-γ (IFN-γ) is considered to be an antagonist of RANKL and is a well-known potent inhibitor of osteoclast function and formation.(39–42) IFN-γ also exhibits antiproliferative actions on primary osteoblast cells.(43–45) Finally, it has been reported that in cultured osteoblasts, AMPK influences the expression of cyclooxygenase 2 (COX-2), which potentially could impact fracture healing because COX-2 is a crucial mediator in mechanically induced bone formation.(46)

In addition to highlighting the potential contribution of the ROA pathway to human wrist BMD deviation, this study also demonstrates the important advantage of pathway-based GWA analysis. None of the genes in the ROA pathway reached a significant level when considered separately; thus the potential impact of the ROA pathway on wrist BMD would not have been detected by individual gene/SNP GWA analyses. By considering critical information about the interaction of a set of functionally related genes and their joint effects, pathway analysis may be a useful paradigm for revealing the polygenic nature of complex diseases. Recently, some candidate pathway association studies have been done, suggesting that the joint actions of common gene variants within pathways may play a major role in predisposing to complex diseases. Examples include the axon guidance pathways for Parkinson disease (PD)(47) and amyotrophic lateral sclerosis (ALS).(48) Differing from the candidate pathway strategies,(47,48) in the current genome-wide analysis we systematically studied a large number of pathways annotated by public pathway databases with the aim of identifying pathways associated with UD BMD. This hypothesis-free strategy could make full use of GWA data and would be more useful for discovering new disease-related processes and generating novel hypotheses about pathogenic mechanisms of disease.

It would be interesting to clarify the detailed mechanism of the ROA pathway on wrist BMD. Considering the existing evidence linking some ROA pathway genes with osteoblastogenesis or osteoclastogenesis, it can be imagined that the involvement of autophagy in bone may not be limited to chrondrocytes alone. Since autophagy is another mechanism for programmed cell death and shows close interaction with apoptosis, whether antophagy is involved in the survival control of osteoblasts/osteoclasts/osteocytes is an important hypothesis to test. Also, it will be interesting to study whether some lifestyle factors will interact with the ROA pathway or if the ROA pathway would be a candidate target for osteoporosis therapy.

In this study we found that the ROA pathway is associated with human wrist BMD variation. However, we cannot exclude other pathways for their significance in wrist BMD variation, even though these pathways did not pass the significance criteria adopted in this study. Regarding the statistical aspect of this study, it should be noted that some genes inevitably are shared by different pathways. Although the overlap of genes among different pathways will not affect the pathways' relative ranking in terms of *NES* values, interdependence between pathways will lead to a decreased power by affecting *FDR* and *FWER* when the causal genes are shared by multiple pathways (owing to the permutation procedure for multiple-testing adjustment). A larger sample size or more specifically annotated pathways will be helpful to improve the power of this pathway-based GWAS.

It also should be noted that under the significance criteria adopted in this study, the ROA pathway was not detected to be associated with hip or spine BMD in the discovery cohort (data not shown). The possible reasons for the inconsistent association signals detected at these three skeletal sites may include statistical errors (type I error at the wrist or type II error at the hip/spine) and genetic heterogeneity between different skeletal sites. In particular, type II error at the hip/spine may be the major reason. The type II error may be caused by the relatively low statistical power of this newly developed pathway-based method (as discussed earlier). This low statistical power, when complicated by the possible genetic heterogeneity (ie, smaller effects of the ROA pathway on hip and spine than on wrist), may lead to negative association signals at the hip and spine.

In summary, we applied a novel pathway-based GWA analysis method to systematically screen functional pathways/gene sets associated with wrist UD BMD and thus osteoporosis. The significant enrichment of ROA pathway genes among the top-ranking genes associated with wrist UD BMD, together with the pathway's functional relevance to bone metabolism, strongly supports an important role of autophagy in human wrist BMD variation. Further detailed and specific functional studies of the ROA pathway will provide new insights into the pathway's relevance to the physiology of bone and the etiology of wrist osteoporosis.
