# Supplementary figures and images for: Pathway-Based Genome-Wide Association Analysis Identified the Importance of Regulation-of-Autophagy Pathway for Ultradistal Radius BMD
Source: J Bone Miner Res. 2010 Jan 29;25(7):1572–80. doi: 10.1002/jbmr.36 (PMC3153999; doi:10.1002/jbmr.36)

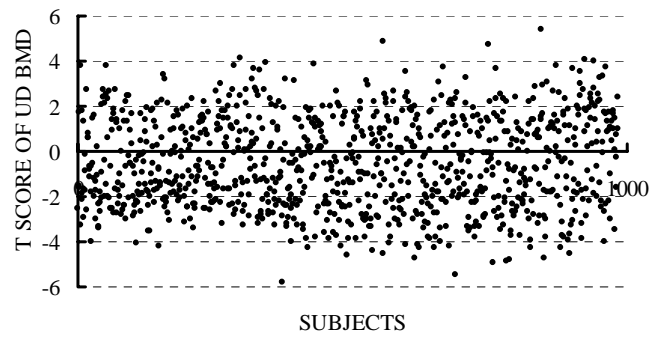

FIGURE S1.

Supplement: Supplementary file 1 [file jbmr0025-1572-SD1.pdf]
