# Supplementary material for: Pathway-Based Genome-Wide Association Analysis Identified the Importance of Regulation-of-Autophagy Pathway for Ultradistal Radius BMD
Source: J Bone Miner Res. 2010 Jan 29;25(7):1572–80. doi: 10.1002/jbmr.36 (PMC3153999; doi:10.1002/jbmr.36)
Supplement: Supplementary file 2 [file jbmr0025-1572-SD2.doc]

TABLE S1. THE LEADING EDGE GENES OF THE ROA PATHWAY IN FHS COHORT

| **Gene** | **SNP^a^** | **P value** | **Role** | **Allele^b^** | **MAF^c^** |
| --- | --- | --- | --- | --- | --- |
| PIK3C3 | rs574957 | 1.97×10^-3^ | Downstream | A/G | 0.488 |
| ATG12 | rs17398382 | 8.16×10^-3^ | Downstream | C/T | 0.157 |
| PRKAA2 | rs2404986 | 8.28×10^-3^ | Upstream | T/C | 0.478 |
| ATG5 | rs2299863 | 1.45×10^-2^ | Intron | T/G | 0.124 |
| GABARAPL1 | rs4764332 | 3.44×10^-2^ | Downstream | G/T | 0.037 |
| BECN1 | rs10512488 | 4.45×10^-2^ | Intron | G/A | 0.231 |
| IFNA13 | rs597408 | 4.72×10^-2^ | Downstream | A/G | 0.061 |
| ATG7 | rs2454511 | 4.92×10^-2^ | Intron | C/T | 0.151 |

^a^ The most significant SNP mapped to each leading edge gene.

^b^ The second allele represents the minor allele of each locus.

^c^ MAF (minor allele frequency) calculated in FHS sample by FBAT software.
